# Application of Chimeric Antigen Receptor T Cells in the Treatment of Hematological Malignancies

**DOI:** 10.1155/2020/4241864

**Published:** 2020-10-01

**Authors:** Weiqi Yan, Zhuojun Liu, Jia Liu, Yuanshi Xia, Kai Hu, Jian Yu

**Affiliations:** ^1^Beijing Advanced Innovation Center for Biomedical Engineering, Beihang University, Beijing 100083, China; ^2^School of Biological Science and Medical Engineering, Beihang University, Beijing 100083, China; ^3^Department of Hematology, Beijing Boren Hospital, Beijing 100070, China

## Abstract

T cell immune protection plays a pivotal role in the treatment of patients with hematological malignancies. However, T cell exhaustion might lead to the possibility of immune escape of hematological malignancies. Adoptive cell therapy (ACT) with chimeric antigen receptor T (CAR-T) cells can restore the activity of exhausted T cell through reprogramming and is widely used in the treatment of relapsed/refractory (r/r) hematological malignancies. Of note, CD19, CD20, CD30, CD33, CD123, and CD269 as ideal targets have shown extraordinary potential for CAR-T cell therapy and other targets such as CD23 and SLAMF7 have brought promising future for clinical trials. However, CAR-T cells can also produce some adverse events after treatment of hematological malignancies, such as cytokine release syndrome (CRS), neurotoxicity, and on-target/off-tumor toxicity, which may cause systemic immune stress inflammation, destruction of the blood-brain barrier, and even normal tissue damage. In this review, we aim to summarize the composition of CAR-T cell and its application in the treatment of acute lymphocytic leukemia (ALL), chronic lymphocytic leukemia (CLL), non-Hodgkin's lymphoma (NHL), Hodgkin's lymphoma (HL), multiple myeloma (MM), and acute myeloid leukemia (AML). Moreover, we will review the disadvantages of CAR-T cell therapy and propose several comprehensive recommendations which might guide its development.

## 1. Introduction

Over the last few decades, the treatment of hematological malignancies has gained tremendous headway, but these diseases still have high morbidity and mortality [1–3]. Traditionally, the treatment of hematological malignancies is administrated by chemotherapy, radiotherapy, and stem cell transplantation. In recent years, with the increased understanding of the molecular genetic basis of these malignancies, immune-targeted therapy has become a new possibility for the treatment of hematological malignancies. Novel understanding in the interaction between immune system and cancer cells of the patient holds great promise for immunotherapy development [4–6].

It is noted that T cell has great potential for immunotherapy of hematological malignancies. The most active T cell endogenous inhibitory pathway is the immunoglobulin superfamily such as CD28/cytotoxic T lymphocyte antigen-4 (CTLA-4): B7-1/B7-2 receptor/ligand grouping, which plays a central role in coordinating immune responses [7, 8]. However, from the beginning of puberty after thymus degeneration, human cytomegalovirus (HCMV) persists due to the chronic activation of cytomegalovirus in humans causing repeated activation of T cells, which is considered the driving factor of human immune aging [9]. Various biological processes including immunoreaction of infection, tumor prevention, and human aging could cause telomere damage, tumor-related stress responses, and T regulatory (Treg) cells activation, which even trigger T cell senescence showing the distinctive phenotypic and functional alternation [9, 10].

Currently, three main strategies are applied to restore the activation of senescent T cells, including replacement, restoration, and reprogramming. Removing senescent T cells from the physiological cycle to maintain the homeostasis of memory and effector T cells is the way of replacement. One method is to target senescent T cells which have selective apoptosis function. Recently, a study showed that FOXO4/p53 was interfered by an engineered peptide, which caused targeted apoptosis of the senescent fibroblasts [11]. In addition, the isolation and storage of umbilical cord blood hematopoietic stem cells have been used to rebuild the immune system for the treatment of hematological malignancies and might be promising for the dynamic equilibrium expansion of functional T cells [12]. Restoration aims to reverse the degeneration of thymus by the combination of bioengineered thymus organoids and growth-promoting factors or cytokines such as IL-21, which may restore and stabilize the thymus environment. Reprogramming is a promising treatment, which redifferentiates T-induced pluripotent stem cells (T-IPSCs) into naïve and cytotoxic T cells or dedifferentiates within their own lineage [13]. Moreover, reprogramming can extend cell lifespan and prevent telomere-dependent T cell senescence by enhancing telomerase activity and telomere length recovery, and thus potentially be used to reverse T cell senescence [14].

Adoptive cell therapy (ACT) as a method of reprogramming is used to recover the activation of senescent T cells by acquiring immune cells from patients or volunteers, performing gene editing and *in vitro* expansion, followed by reinjecting them into patients which is extensively applied in the treatment of T cell senescence [15]. The sufficient effector cells with antitumor recognition capabilities from patients improve the effectiveness of ACT [15]. As a type of ACT, CD-19-targeted CAR-T cell therapies have shown dramatic results for the treatment of hematological malignancies, which was approved by the US Food and Drug Administration (FDA) [16].

## 2. CAR-T Cell

**Figure 1:**
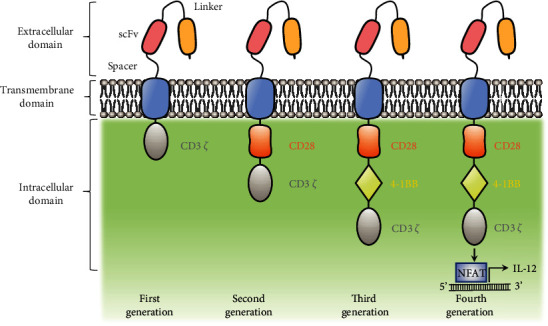
The general structure of CARs of different generations.

CAR-T cell technology integrates the chimeric antigen receptor gene into the patient's T cells through genetic engineering. Specifically, T cells are extracted from patients' blood or tumor tissues, followed by applying genetically engineering and transfection *in vitro* to produce a certain number of CAR-T cells, which are finally reinjected into tumor patients to induce antitumor cell action. The structure of CAR is a hybrid molecule, which mainly includes three parts: extracellular domain, transmembrane domain, and intracellular domain (see Figure 1). The extracellular domain is a single chain variable fragment (scFv) that is responsible for surface antigen recognition based on the structure of monoclonal antibodies. The transmembrane domain mainly serves to maintain the CAR structure, and the intracellular domain is composed of the T cell receptor (TCR), which activates the signal pathways required by T cells [17]. At present, the design of CAR-T cell has mainly experienced four generations of development. The intracellular structural design of the first-generation CAR is mainly composed of CD3*ζ* that is connected to a direct, independent MHC antigen recognition domain for antigen on the surface of the tumor cells, which is similar to endogenous TCR complex [18]. However, the design of the first-generation CAR-T cells do not have a costimulatory signal domain, providing T cells with full activation of the second messenger, which leads to their limited potential for expansion *in vivo*, poor cytotoxicity on tumor cells, and more prone to apoptosis. To make up for the shortcoming of the first-generation CAR, the researchers add a costimulatory signal domain (such as CD28 and 4-1BB) to improve the signal activation ability and the viability of T cells, which could induce an effective expansion of the modified T cells *in vivo* [18]. In fact, the structural design of the second-generation CAR has laid the foundation for the currently approved CAR-T cell therapy. Nevertheless, the current study shows that the role of different types of costimulatory domains in CAR signaling is specific. For example, CD28-based CAR-T cells exhibit more powerful effector cell functions and limited durability [19], but the 4-1BB tends to drive CAR-T cells to the development of central memory phenotype to improve durability [20]. Similarly, the design of the third-generation CAR-T cells combines the signal potential of two costimulatory signal domains (such as CD28 and 4-1BB) to fill the gap of the second generation [21]. The fourth-generation CAR-T cells increase the anti-tumor activity by further enhancing additional genetic modifications. It is engineered with an activation inducible element such as a nuclear factor of the activated T cell (NFAT)-responsive expression cassette to facilitate secretion of transgenic cytokines such as interleukin-12 (IL-12), interleukin-15 (IL-15), and interleukin-18 (IL-18), which are known as T cell redirected for universal cytokine-mediated killing (TRUCK) [22]. IL-12 recruits additional immune cells to fight cancer cells which are not identified by CAR-T cells [23]. Moreover, IL-12 is related to the proliferation, terminal differentiation, and exhaustion of T cells [24]. Conversely, IL-15 maintains the homeostasis of CD8^+^ memory T cells, enhances the antitumor activity, and reverses T cell senescence [24]. Furthermore, some researchers demonstrated that CAR-T cells expressing IL-18 exhibited continuous antitumor activity [25]. In general, these immune functional modifications exhibit great antitumor effect in fourth-generation CAR-T cells.

## 3. CAR-T Cells for Hematological Malignancies

### 3.1. Acute Lymphoblastic Leukemia

Acute lymphoblastic leukemia (ALL) is a common hematological malignancy caused by abnormal bone marrow blasts and naïve cells with excessive proliferation. CAR-T cells are used in the treatment of ALL, especially the lethal relapsed/refractory (r/r) B-ALL [26]. CD19 is an activated transmembrane glycoprotein regulating B cells in an antigen receptor-dependent manner, which is expressed throughout the differentiation process of B cells, especially during the malignant transformation of B cells [27]. Based on this, CD19 as an ideal target for the treatment of lymphomas is attractive to researchers. Grupp et al. applied anti-CD19 antibody and T cell signaling molecule CTL019 CAR-T cells to treat 2 patients with r/r ALL. It was revealed that the level of CTL019 CAR-T cells expansion in the two patients was 1000 times than the original transplantation level by bone marrow examination. In addition, CAR-T cells were also observed to maintain high levels for at least 6 months in cerebrospinal fluid (CSF), and both patients were observed complete remission (CR) [28]. Furthermore, Maude et al. used tisagenlecleucel (formerly CTL019) to treat patients with r/r B cell ALL. 75 patients were injected with tisagenlecleucel, and the complete remission rate (CRR) within 3 months achieved 81%. All patients who responded to the treatment were evaluated by flow cytometry, and the minimal residual disease (MRD) was negative. The duration of tisagenlecleucel in the blood was observed for up to 20 months [29]. Moreover, a study reported 16 cases of r/r B-ALL patients treated with autologous T cells expressing CD19 antigen-specific 19-28Z CAR. The overall CRR achieved 88%, according to IgH deep sequencing evaluation and morphological criteria [30]. However, because leukemia cells exhibit the same antigen as normal T cells, the identification of targets for ALL cells poses challenges. Especially, CAR-T cell therapy for ALL always presents cytokine release syndrome (CRS) [31].

### 3.2. Chronic Lymphocytic Leukemia

Chronic lymphocytic leukemia (CLL) is the most common type of adult leukemia, and patients with multiple r/r CLL have a poor prognosis [32]. It is an effective approach to treat CLL by targeting CD19 with CAR-T cells. A team of researchers at the University of Pennsylvania designed the CAR-T cell that can bind to CD3zeta (a signal-transduction component of the T cell antigen receptor) and CD137 (a costimulatory receptor in T cells). After low-dose (about 1.5 × 10^5^ cells per kilogram of body weight) autologous CAR-T cells were injected into CLL patients, it was found that the number of CAR-T cells expanded 1,000 times than the initial input, and the tumor was completely relieved. In addition, the CAR was expressed continuously in the blood and bone marrow for 6 months [33]. Furthermore, this team also reported the results of the CAR-T cell therapy of 14 patients with r/r CLL. They infused CTL019 T cells at 0.14 × 10^8^ − 11 × 10^8^ (median 1.6 × 10^8^ cells) to patients with r/r CLL and monitored the circulating CTL019 T cells for toxicity, responsiveness, expansibility, and persistence. It was indicated that the total effective rate of these treated CLL patients was 8/14 (57%), including 4 CRs and 4 partial remissions (PRs) [34]. Recently, researchers have used other therapies combined with CAR-T cell therapy for CLL patients to show a certain effect. Geyer et al. reported that CD19-targeted CAR-T cells integrated with CD28 intracellular signaling domain (19-28Z) can be a consolidation therapy for the treatment of 8 CLL patients after first-line chemotherapy of infliximab. 3 of 8 patients (38%) achieved objective response (OR), from which the clinical CR of 2 patients sustained more than 28 months. 4 patients developed self-limiting fever after infusion of CAR-T cells, while interleukin-6 (IL-6), interleukin-10 (IL-10), interleukin-2 (IL-2), and transformation growth factor-*α* (TGF-*α*) were elevated. CAR-T cells were detected in 4 patients after infusion, and the longest duration was 48 days [35]. Further, Gauthier et al. reported 19 cases of r/r CLL patients treated with CAR-T cells and ibrutinib, discovering that the 4-week CRR was 83% and 61% of patients attained MRD by IgH sequencing negative bone marrow response. In the cases with or without ibrutinib, the 1-year PFS after CAR-T cell therapy was 38% and 50%, respectively. The simultaneous application of ibrutinib and CAR-T cell therapy was well tolerated [36]. These studies indicate that CAR-T cells are effective in the treatment of CLL.

### 3.3. Non-Hodgkin's Lymphoma

Non-Hodgkin's lymphoma (NHL) is also one of the most common B cell lymphomas. Recently, although chemotherapy, radiotherapy, and hematopoietic stem cell transplantation have gained significant progress in the treatment of NHL, the mortality has not declined. For patients who are resistant to standard treatment regimens, new treatment approaches are imperative. Recently, CAR-T cell therapy has received widespread attention due to its remarkable success in r/r lymphoma [26].

Diffuse large B cell lymphoma (DLBCL) is the most common subtype of NHL and has an aggressive clinical course. A study reported that CAR-T cells expressing anti-CD20 were used to treat two patients with relapsed DLBCL after autologous hematopoietic stem cell transplantation; no clinical responses and no obvious toxicities were observed [37]. Coincidentally, Kochenderfer et al. reported the application of anti-CD19 CAR-T cells in 15 patients with advanced B cell malignancies, from which, 4 of 7 chemotherapy-refractory DLBCL patients achieved CR [38]. Further, the SCHOLAR-1 study analyzed the efficacy and survival results of 636 patients with r/r DLBCL retrospectively. The objective response rate (ORR), CRR, and median overall survival (OS) of the r/r groups were 26%, 7%, and 6.3 months, respectively [39]. Moreover, Schuster et al. reported 28 lymphoma patients in adult received CTL019 CAR-T cell therapy, and 6 of 14 cases of DLBCL had CR [40]. Furthermore, Stirrups and colleagues used anti-CD19 CAR-T cells to treat patients with refractory large B cell lymphoma. They recruited 111 patients with histologically confirmed large B cell lymphoma, including DLBCL and primary mediastinal B cell lymphoma, and autologous anti-CD19 CAR-T cells were inoculated at 2 × 10^6^ cells/kg body weight. Of the 101 patients included in the intention-to-treat analysis, 83 patients had OR with CR in 55 cases (54%) and PR in 28 cases (28%) [41].

In addition, mantle cell lymphoma (MCL) accounts for 7% in all NHL [42]. CAR-T cell therapy is an effective approach for the treatment of MCL, and it is possible to make MCL a curable disease. Till et al. conducted a preclinical trial in 4 patients with relapsed indolent B cell and MCL, evaluating the effect of the third-generation CD20-specific CAR-T cells with CD28 and 4-1BB costimulatory domains. According to the result, this therapy was well tolerated, although transient infusion symptoms were tested in 1 patient. No progress was continuous in 2 patients at 12 and 24 months, but an objective PR occurred in another patient, and the disease relapsed 12 months after injection [43].

Additionally, some researchers also applied CAR-T cells for the treatment of other subtypes of NHL. Schuster et al. applied CTL019 in a Phase IIa study of 14 follicular lymphoma (FL) patients, suggesting that they had disease progression within 2 years after treatment with 2 or more treatment lines [40]. In addition, Neelapu et al. treated refractory and aggressive B cell NHL patients with KTE-C19 consisting of a single-chain antibody called FMC-63 in the extracellular region, which recognize CD19 on the surface of tumor cells. After treatment, it was found that 62 patients have a total effective rate of 79% and a complete effective rate of 52% [44]. Besides, Chen et al. reported a case of r/r acute B cell lymphoblastic lymphoma (B-LBL) with Li-Fraumeni syndrome (LFS), receiving anti-CD19 and anti-CD22 CAR-T cell “cocktail” treatment. The morphology and multiparameter flow cytometry showed that the tumor was completely relieved, and the MRD was negative [45]. It was worth noting that Burkitt lymphoma (BL) was also one of the most common subtypes of NHL in children, and 5%-10% of patients with a poor prognosis still relapsed after intensive chemotherapy. Recently, Du et al. treated an eight-year-old boy with CAR-T cells targeted to the antigens CD19, CD22, and CD20 sequentially. The results showed that the child had no obvious response to anti-CD19 CAR-T cell therapy and showed progressive disease (PD). After CAR-T cell therapy guided by CD22, the child experienced PR but unfortunately relapsed quickly. Finally, after receiving anti-CD20 CAR-T cell therapy, the child achieved CR [46].

In addition, CD23 and orphan tyrosine kinase receptor (ROR1) have also become potential targets for the treatment of B cell lymphomas, and CAR-T cell therapy has shown great promise in the advancement of the r/r NHL therapy [26].

### 3.4. Hodgkin's Lymphoma

Hodgkin's lymphoma (HL) is a hematological malignancy originating from B cells and overexpresses CD30 which is a potential therapeutic target [26]. According to a Phase I study, 7 patients with r/r HL were treated with anti-CD30 CAR-T cells, 1 case achieved CR for more than 2.5 years, 1 case sustained CR for almost 2 years, and 3 cases had transient stable disease (SD). It was a remarkable fact that the anti-CD30 CAR-T cell therapy had no toxicity in this study [47]. Furthermore, Wang et al. performed anti-CD30 CAR-T cell therapy on 11 patients with r/r HL, infusing 1.5 × 10^7^ CAR-T cells per kg of weight. It was indicated that 9 cases (82%) responded to treatment, 1 case (9%) maintained continuous CR, 1 case (46%) achieved PR, and 3 cases (27%) were stable. Tolerable infusion-related fever syndrome was occurred in all patients. One patient (9%) had self-limited arthralgia, myalgia, and swelling of both knees for 5 days after 2 weeks of infusion [48]. These results confirm that CD30 has great potential in the treatment of HL.

### 3.5. Multiple Myeloma

Multiple myeloma (MM) is a refractory malignancy of bone marrow origin, which is caused by a malignant mutation of plasma cells forming in the final stage of B cell development [49]. CD19 is hardly expressed on the surface of MM cells, and the killing effect of anti-CD19 CAR-T cells on MM cells is weak, which even damages some healthy tissues [50]. Therefore, searching for specific targets expressed on MM cells but not on healthy tissue cells is a potentially effective method for the treatment of MM. Hosen et al. found a specific therapeutic target (active conformation of integrin) for the treatment of MM. By screening more than 10,000 anti-MM monoclonal antibodies, they identified MMG49 as the MM monoclonal antibody recognizing the integrin *β*7 molecule specifically. It was therefore evident that the transduced MMG49-derived CAR-T cells played an anti-MM role without damaging normal hematopoietic cells [51]. Furthermore, B cell maturation antigen (BCMA, CD269) is another identified target molecule for the treatment of MM. Xu et al. reported a female patient with r/r MM after infusion of anti-BCMA CAR-T cells achieved CR. However, patients with a disease-free survival of 7.6 months developed grade 1 CRS which was mainly manifested as fever and nausea and eventually led to the relapse of MM [52]. Similarly, CD138 (also known as syndecan 1) is highly expressed on the surface of both normal and malignant plasma cells, which has been identified as an attractive target for a long time. Sun et al. found that T cells from healthy donors and patients with MM could eliminate MM cell lines and primary myeloma cells *in vitro* and *in vivo* when transduced with CD138-specific chimeric antigen receptors. Through preclinical analysis, no off-target tumor cytotoxicity occurred in normal epithelial or endothelial cells [53]. Further, they used SLAMF7 as the target to prepare CAR from anti-SLAMF7 antibody huLuc63 (elotuzumab), indicating that these CAR-T cells had a rapid lysis effect against MM cell lines and primary MM cells *in vitro* [54]. All in all, CAR-T cell therapy for MM has become a promising strategy.

### 3.6. Acute Myeloid Leukemia

Acute myeloid leukemia (AML) is a hematological malignancy with high relapse rate in adults and children [55, 56]. In this regard, some studies have shown that the application of CAR-T cells to treat AML has achieved significant effects. A research team showed an anti-CD123 CAR-T cell therapy for the treatment of AML [55]. According to the initial *in vitro* study, anti-CD123 CAR-expressing cytokine-induced killer T cells demonstrated great antitumor activity [55]. Moreover, a method for preparing allogeneic CAR-T cells by targeting CD33/Siglec-3 antigen from healthy donors has been developed [57]. Anti-CD33 CAR-T cells reduced AML tumors effectively in mouse model trials [57]. Furthermore, FMS-like tyrosine kinase 3 (FLT3) was discovered as a novel target for CAR-T cell therapy in AML [58]. FLT3-inhibitor crenolanib and FLT3 CAR-T cells demonstrated synergistic antileukemia effects in mouse trials [58]. In addition, John et al. demonstrated that leukocyte immuno-globulin-like receptor-B4 (LILRB4) was highly expressed on the surface of AML cells [56]. They constructed anti-LILRB4 CAR-T cells successfully, which had a specific recognition effect on AML cells [56]. Although the above CAR-T cell therapy targets for AML have not been used in clinical trials, they provide hope for the further development of treatment for AML.

## 4. Disadvantages of CAR Treatment

### 4.1. Cytokine Release Syndrome

CRS is a systemic immune stress inflammation, which is caused by the rapid proliferation and release of cytokines after the introduction of CAR-T cells into the patient. It is one of the most common adverse events in CAR-T cell therapy. It usually exhibits symptoms such as hypotension, fever, neurological changes, and hypoxia [59]. According to the above experimental results, anti-CD19 CAR-T cells are effective in treating hematological malignancies, but they also produce a large number of cytokines, resulting in CRS in most patients after treatment. At present, many researchers have designed new CAR-T cell therapy programs for CRS. A research team determined the diagnostic criteria for severe cytokine release syndrome (SCRS) through clinical analysis of 21 days after T cell infusion and systemic analysis of serum cytokine levels, with the aim of better identifying patient subgroups which may require corticosteroids or IL-6 receptor blockers for therapeutic intervention to suppress CRS. In addition, they also found that serum C-reactive protein (CRP) could be applied as a reliable standard to evaluate the severity of CRS, paving a way for patient management in centers currently considering CAR-T cell therapy [30]. Tocilizumab is a humanized anti-IL-6 receptor (IL-6R) monoclonal antibody approved by the FDA for the treatment of SCRS. A study has shown that CRS subsides rapidly after taking tocilizumab and will not adversely affect CAR-T cell therapy [60]. Caimi et al. found that the application of prophylactic tocilizumab (PT) before injecting anti-CD19 CAR-T cells with CD3zeta/4-1BB costimulatory domain could reduce the incidence of SCRS [61]. Moreover, Jiang et al. found that CRS caused by CAR-T cell therapy would further lead to disseminated intravascular coagulation (DIC) and proposed the application of immunosuppressive agents and corticosteroids for early and appropriate interventions for CRS-related coagulation disorders, which could be used to control the side effects of CAR-T treatment [62].

### 4.2. Neurotoxicity

Neurotoxicity is the second most severe adverse event of CAR-T cell therapy. Typical manifestations of neurotoxicity include mild headache, language disorder, visual hallucination, seizure, severe encephalopathy, and even death [63]. Neurotoxicity is related to CRS, which can occur alone or with CRS after CAR-T cell therapy, but the mechanism leading to neurotoxicity is not yet clear. Based on data from patients and animal models, Gust et al. reported that damage to the blood-brain barrier (BBB) in patients is related to high levels of cytokines and endothelial activation in the blood and cerebrospinal fluid. Patients with neurotoxicity were often treated by corticosteroids, IL-6 targeted therapy, and supportive care, but their effectiveness needed to be proved [64]. Further, they also provided evidence of severe neurotoxicity showing endothelial activation in 133 patients with refractory B cell malignancies treated with anti-CD19 CAR-T cells, including capillary leak, diffuse intravascular coagulation, and increased BBB permeability [65]. A study showed that after CAR-T cell therapy of patients with r/r DLBCL, the level of IL-15 and granulocyte-macrophage colony-stimulating factor (GM-CSF) was increased, which was associated with severe neurotoxicity [66]. Sterner et al. showed that neutralizing GM-CSF could prevent CRS and reduced neurotoxicity significantly, and this method reduced neuroinflammation and BBB damage by 75% and enhanced antitumor activity [67]. Recently, Harden et al. analyzed 12 plasma samples of adult patients with varying degrees of neurotoxicity after CAR-T cell therapy, suggesting that the levels of apolipoprotein A1 (ApoA1) and angiogenin changed over time, which might indicate the risk of neurotoxicity. This correlation offers great promise in the clinical treatment of neurotoxicity research and development, but it needs to be verified by quantifying the absolute concentration of ApoA1 and angiogenin in the blood of a larger patient population [68].

### 4.3. On-Target/off-Tumor Toxicity

In addition to tumor tissues, biomolecular markers of hematological malignancies are also expressed in some normal tissues, especially lymphoid tissues. Although CAR-T cells have relative specificity, each scheme may cause different degrees of tissue damage called on-target/off-tumor toxicity [69]. Comprehensive treatment based on the combined control system of switchable dual receptor CAR-T cells and immune checkpoint blockade has become a new paradigm for CAR-T cell therapy [70]. Wendell Lim's laboratory has developed a system that requires two antigens to fully activate CAR-T cells, called synNotch, consisting of an engineered antigen recognition domain, an artificial transcription factor (ATF), and a Notch core. Among it, the ATF is activated under antigen stimulation to induce CAR expression specifically. Intermediate signal molecules are not required in this system which works in an orthogonal manner. However, whether the ATF is immunogenic remains to be studied [71–73]. Moreover, Fedorov et al. designed an inhibitory CAR (iCAR) therapy that could coexpress and recognize antigens expressed in nontumor tissues. iCAR is connected to the intracellular domain of T cell checkpoint protein by an antigen recognition domain, named cytotoxic T lymphocyte associated protein 4 (CTLA-4) or programmed cell death protein 1 (PD-1). Although cells expressing iCAR targets have activated CAR antigens, they will not activate T cells to avoid damage to normal tissues [74]. These methods not only ensure the antitumor activity of CAR-T cells but also prevent off-tumor toxicity.

### 4.4. Relapse with CAR-T Cell Therapy

Although most patients after receiving CAR-T cell therapy would achieve high CR, relapse of hematological malignancies may occur [75]. One cause of relapse is the loss of antigen after CAR-T cell therapy, which is also called target-negative relapse [75]. Additionally, a study has shown that changes in the CAR-T cell microenvironment may induce antigen-positive relapse [28]. Searching novel targets and allogeneic hematopoietic stem cell transplantation (allo-HSCT) are the main strategies for the treatment of relapse [26].

## 5. Future Direction

In recent decades, CAR-T cell offers tremendous progress in the treatment of hematological malignancies, but there are still many challenges. The problems mainly include how to improve the effectiveness and durability of CAR-T cells in patients and how to alleviate the adverse events after treatment [69]. A novel bispecific CAR targets two tumor-specific markers, leading to better therapeutic efficacy [69]. A study reported that a functional CAR called TanCAR was designed to mediate bispecific activation of T cells [76]. It was suggested that TanCAR induced T cells reactivity against two different antigens and produced synergistic enhancement of T cell activation [76]. In terms of achieving the durability of CAR-T cell therapy, there have been reports of using oncolytic viruses with chemokine genes to recruit CAR-T cells for the treatment of solid tumors. Oncolytic viruses have the ability to prolong their duration and directly attack tumor cells [77, 78], which might have great potential to treat hematological malignancies. Moreover, the development of universal CAR-T cell therapy for the treatment of hematological malignancies can avoid the problem that patients' autologous T cells are difficult to obtain and reduce the cost of treatment. We hypothesize that the application of CRISPR technology to edit allogeneic genes provides potential for the development of universal CAR-T cell therapy.

For the adverse events generated after CAR-T cell therapy, the main therapeutic drugs include tocilizumab and corticosteroids, which have been mentioned specific cases in the above. Furthermore, Laborda et al. summarized an approach to activate predesigned cell triggers allowing CAR-T cells to be eliminated in case of adverse events in patients [79]. For example, some researchers designed epitope markers expressed by the CAR vector in the form of cistrons and approved that monoclonal antibodies could then eliminate the labeled cells [79]. This strategy has been used to reverse the anti-CD19 CAR-T cell-mediated B cell aplasia in mice, which has shown great promise of the elimination for patients' CAR-T cells after a period of remission [80]. Another method called “suicide gene system” is also worthy of attention [81]. Chemically induced caspase 9 (iCas9) dimerization is one of the most prominent suicide genes in T cell based immunotherapy, which has shown extraordinary efficiency in clinical trials of patients [82]. In general, these therapies might avoid the adverse events caused by CAR-T cells for secondary drug treatment, which also show a new perspective for future direction.

## 6. Conclusion

CAR-T cells have shown great potential in the treatment of hematological malignancies such as ALL, CLL, NHL, HL, MM, and AML. According to different types of hematological malignancies, it is the basic idea of this therapy to screen out specific targets on the surface of the respective tumor cells and construct CAR-T cells *in vitro* to be imported into patients. CAR-T cells have been applied in clinical treatment for a long time and have gained headway in the field of hematological malignancies. However, there are still many disadvantages, such as CRS, neurotoxicity, off-tumor toxicity, and relapse. The development of new CAR-T cell therapies to compensate for or alleviate these deficiencies is the great responsibility of the researchers.
